# Seasonal Dynamics and Physiological Age of Ixodid Ticks Collected from Dogs

**DOI:** 10.3390/ani13193026

**Published:** 2023-09-26

**Authors:** Aleksandra Petrović, Ksenija Stanić, Aleksandra Popović, Ivana Ivanović, Dejan Supić, Dušan Marinković, Vojislava Bursić

**Affiliations:** 1 Faculty of Agriculture, University of Novi Sad, Trg Dositeja Obradovića 8, 21000 Novi Sad, Serbia; aleksandra.petrovic@polj.edu.rs (A.P.); ksenija.stanic@gmail.com (K.S.); ivana.ivanovic@polj.edu.rs (I.I.); dusan.marinkovic@polj.uns.ac.rs (D.M.); vojislava.bursic@polj.uns.ac.rs (V.B.); 2 Agro-Vet, Šenoina 16/I, 24000 Subotica, Serbia; 3 Faculty of Ecological Agriculture, University Educons, Vojvode Putnika 87, 21208 Sremska Kamenica, Serbia; dejan.supic@educons.edu.rs

**Keywords:** ticks, dogs, morphometric characteristic, body mass, scutal index, physiological age

## Abstract

**Simple Summary:**

A large number of dog owners are informed about ticks and tick-borne diseases that could be dangerous for their pets. However, they are not familiar with tick biology, seasonal activity, and the possibility that some pathogens could be transmitted from dogs to humans. The aims of this study were to determine tick species and their seasonal occurrence on pet dogs and to compare the accuracy of three indices frequently used to calculate tick female physiological age in terms of feeding process duration. As a result of a one-year study, four tick species were identified and their seasonal patterns showed a continuous occurrence on dogs through the whole year, even during the winter months. The most accurate index was the physiological age index, the only tested index which included body mass as a variable measure. Seasonal activity of ticks depends on numerous factors: abiotic, biotic, and anthropogenic. Tick species diversity and abundance in specific geographical range varied in recent years, due to global climate changes and increase of human and animal migrations. These topics demand the One Health approach, where the integrated efforts of scientists, veterinarians, and physicians are essential in order to prevent and manage tick-borne pathogens and diseases.

**Abstract:**

In order to reproduce and complete life cycles, ticks have to feed on different hosts, thus participating as vectors and reservoirs in the maintenance and circulation of different pathogens. Since dogs can serve as suitable hosts for numerous tick species, the aims of this study were to determine tick species and their seasonal occurrence on pet dogs and to compare the accuracy of three indices frequently used to calculate engorged female physiological age. Ticks were collected from dogs brought to veterinary clinics. Three indices were analyzed: scutal index, alloscutal/scutal index ratio, and physiological age index. Four tick species were identified: *Ixodes ricinus*, *Dermacentor marginatus*, *D. reticulatus*, and *Rhipicephalus sanguineus* group, and the last was the most abundant. The highest number of collected ticks was in April, but two species were continuously active throughout the year. The statistical analyses distinguished the physiological age index as more precise because of lower variability. Dog owners usually ignore regular dog anti-tick treatments throughout the year, as they are not aware that ticks could be active during the winter months. Tick surveillance is unquestionably important in order to monitor and prevent the distribution of these vectors and also the diseases they transmit.

## 1. Introduction

Ticks are vectors and reservoirs of numerous pathogens such as viruses, bacteria, and protozoa. Occurring in and maintaining a multifunctional and multilevel system, tick–host–pathogen, the transmission of tick-borne pathogens (TBPs) to humans and animals has become more frequent in recent years worldwide [1]. The biology and evolution of the tick–host–pathogen molecular interactions resulted in conflict and cooperation between them [2] and induced divergence in tick vector potential and competence. Therefore, different tick species are capable of transmitting different disease pathogens, which depends on numerous factors, especially their genetic determinants as vectors [3].

Ixodid ticks (family Ixodidae) are obligatory ectoparasites of domestic and wild animals, as well as humans, and have to feed on vertebrate hosts (amphibians, reptiles, birds, and mammals) in order to reproduce and complete life cycles [4]. During hematophagous feeding processes on different hosts, they absorb pathogens and transmit them further, not only through the specific feeding manner but also through transovarial and trans-stadial transmission. Subsequently, if the pathogen is present, the infection of the succeeding host is inevitable [5]. Therefore, ixodid ticks are significant vectors of a broad range of pathogens of medical and veterinary importance on a global scale that can cause tick-borne diseases (TBDs) like borreliosis, babesiosis, anaplasmosis, ehrlichiosis, rickettsiosis, bartonellosis, piroplasmosis, hepatozoonosis, tularemia, tick-borne encephalitis, and different hemorrhagic fevers, such as Crimean-Congo hemorrhagic fever [6].

Dogs (*Canis familiaris* Linnaeus, 1758) can serve as hosts for several tick species, of which the, *Ixodes ricinus*, *Ixodes hexagonus*, *Dermacentor reticulatus*, and *Rhipicephalus sanguineus* groups are the most prominent in Europe [7]. Several studies conducted as nationwide surveys and published from the beginning of this century have reported different tick species infesting dogs in European countries: *Ixodes ricinus* [3,5,6,7,8,9,10,11,12,13,14,15,16,17]; *I. persulcatus* [3]; *I. arboricola* [11]; *I. canisuga* [5,6,8,11,13,14,17]; *I. gibbosus* [11]; *I. festai* [11]; *I. acuminatus* [14]; *I. hexagonus* [5,6,7,8,9,10,11,13,14,15,16,17]; *D. marginatus* [5,6,11,18]; *D. reticulatus* [3,5,6,7,8,9,10,11,13,14,16,17,18]; *Haemaphysalis punctata* [5,8,11]; *H. concinna* [6,12,13,14]; *H. parva* [12]; *R. sanguineus* [5,7,8,10,11,12,16]; *R. bursa* [5,11,12]; *R. turanicus* [12,16], and *Hyalomma scupense* [12]. A non-indigenous species for Europe, *Dermacentor variabilis*, was reported in 2016 in the UK on one dog which was imported from the United States of America [8]. The risk of non-indigenous tick species occurrence in Europe, and consequently, new TBPs and TBDs is rising because of evident increase in human and animal migrations, through immigrations, tourism, traveling, transport, and animal trade routes.

Dog-related TBPs are important as some of them can be dangerous also to human health, causing zoonotic diseases [6]. There are several factors that are directly or indirectly involved in increasing the prevalence of TBPs in owned dogs: human population growth, urbanization and habitat changes, human and, therefore, pet dog behavioral changes in terms of more frequent and longer activities in nature where they encounter wildlife, global climate changes, and increased wildlife populations in urban and peri-urban areas [5,7,11]. Therefore, dogs could serve as a means by which infected ticks can be carried into domestic settings, thus enhancing the risk of human infection [5].

As opposed to vertebrates, in which physiological (biological) age is usually considered as a whole life cycle, from birth to death, the physiological age of ticks, and other, especially hematophagous, arthropods is an independent parameter for each of the active life-cycle stages [19]. An adult ixodid tick female requires 6 to 10 days to take a complete blood meal and engorge fully [20] depending on the host species. As a long-term pool feeder, the female ingurgitates all fluids exuded into the hemorrhagic pool which is generated by its bite [21]. Considering the duration of this unique feeding process, it could be divided into three phases: preparation, a slow, and a rapid phase. The first phase could last up to 2 days during which the female creates a feeding lesion, secretes saliva [21] and cement [22], a rapidly hardening substance, which ensures firm and secure attachment and enables long-lasting contact with the host. The slow phase continues next for 4 to 7 days during which a female actively feeds and increases its body mass up to 10 times. The feeding process ends with a rapid phase, which lasts only 24 to 36 h, during which a female rapidly increases its weight more than 10 times [21]. Blood meal ingestion causes a cuticle expansion, which can be observed through a decrease in the depth of the dorsal and lateral grooves on the alloscutum surface. This is caused by the cuticle growth process during both the slow and rapid phase and represents a general characteristic of the hard tick species [23].

The morphometric parameters and, based on them, the calculated indices can be very useful in determining the age structure of the tick population collected from vegetation, as well as identifying the duration of parasitism on the host, i.e., duration of the blood meal adoption [24] and, thus, the potential risk of pathogen transmission [25]. The most frequently used morphometric parameters in measuring a tick’s physiological age are the scutal index, the alloscutal index, body mass, and their ratios [24,25,26,27,28,29,30].

During absorption of a blood meal, the tick alloscutum length and width drastically increase (especially in females), while the scutal shield dimensions remain constant. Consequently, several indices were created in order to describe the physiological age of ticks with the aim to derive quantitative parameters that compare scutal dimensions and other body characteristics in order to calculate the time spent on the host and, therefore, possible TBP transmission [27].

Taking into consideration the importance of dogs as a link between humans and ticks in urban areas, the aims of this study were to determine tick species diversity and seasonal incidences of pet dog infestation, as well as to compare the accuracy of three indices frequently used to calculate the physiological age of engorged females.

## 2. Materials and Methods

### 2.1. Tick Sampling

Ticks were collected during a year’s research period, from January to December in 2020, from veterinary offices in Subotica and Novi Sad (cities in Vojvodina, the northern province of the Republic of Serbia). A total number of 1025 pet dogs of different breeds was inspected visually and with the palpatory method by veterinarians, and all detected ticks were gently detached using tweezers in order to avoid rupture, tearing, or other body injuries. Ticks were stored in plastic vials with perforated stoppers and a small ball of cotton wool soaked in water in order to obtain sufficient air ventilation and moisture. In this way, ticks were preserved live, in good condition and fitness for further analyses and measurement.

### 2.2. Tick Identification and Morphometric Measurement

Ticks were identified up to species level according to the identification keys [31]. Seasonal dynamics were analyzed for nymphs, females, and males of all collected species. Furthermore, 30 partially or fully engorged females of each identified species with intact body were segregated for further morphometric measurements. Morphometric characteristics were measured using a Motic stereomicroscope with a Moticam camera 1000 (Speed Fair, Hong Kong, China). Eight dorsal morphometric characteristics were measured: capitulum base length and width, length between cornua, scutum length and width, alloscutum length and width, and lateral width. The body mass was measured using analytical scale Kern 440-47N (KERN& SOHN GmbH, Balingen, Germany). The morphometric parameters were measured within the first two hours after tick removal.

### 2.3. Physiological Age Calculation

For the purpose of the study, three indices were used.

Scutal index (SI) was calculated as the ratio between alloscutum length (a) and scutum width (b) [26]:

SI = a/b(1)

The alloscutal index/scutal index ratio (ASR) was calculated according to the following equation [27]:

ASR = ((B_l_ × B_w_) − (S_l_ × S_w_))/(S_l_ × S_w_) (2)

where multiplication of B_l_ (total body length) and B_w_ (body width in the widest plane) produced body index. The product of S_l_ (scutal length) and S_w_ (scutal width) multiplication generated scutal index. The subtraction of these values created the alloscutal index. Therefore, ASR was calculated as a ratio between alloscutal and scutal indices.

The physiological age index (PAI) included the third dimension and, therefore, was calculated as

PAI = ∛B_m_/S_l_ (3)

where B_m_ was an individually measured body mass (in grams) and S_l_ was scutal length (in millimeters) [28]. All indices were mathematically calculated using Microsoft Office Excel (Microsoft Office Standard, 2019, University License).

### 2.4. Statistical Analyses

Descriptive statistics parameters such as mean (M), minimum (Min), maximum (Max), variance (V), standard deviation (SD), coefficient of variation (CV), and standard error (SE), as well as analysis of variance (ANOVA) and Fisher’s Least Significant Difference (LSD) test were performed using Statistica 14.0.0.15 (TIBCO Software Inc., Palo Alto, CA, USA, University License).

## 3. Results

Four tick species were identified: *I. ricinus* (Linnaeus, 1758), *D. marginatus* (Sulzer, 1776), *D. reticulatus* (Fabricius, 1794), and *R. sanguineus* group (Latreille, 1806). The total number of collected ticks was 1057. The most abundant species was *R. sanguineus* (64.33%), followed by *D. reticulatus* (21.76%). The less frequently found species were *I. ricinus* and *D. marginatus* (8.52% and 5.39%, respectively) (Table 1). Only the presence of *I. ricinus* and *R. sanguineus* nymphs were noticed (Table 1).

### 3.1. Tick Seasonal Dynamics

More than half of the sampled ticks (650) were collected during April, which might highlight this period as the most perilous month for pet dogs to encounter ticks during the everyday walking routine. However, a more detailed analysis highlighted that 88.46% of this number were *R. sanguineus* specimens. The highest number of *Dermacentor* specimens was observed in March, and *I. ricinus* was most abundant in May (Table 1). *R. sanguineus* was present on examined dogs only six months through the year (from April till September), while the other three species demonstrated a usual bimodal dynamic, similar to one demonstrated in natural conditions (i.e., when questing ticks are sampled from vegetation). Additionally, *D. reticulatus* was present in high number during late autumn and winter months (Table 1) which indicated its plasticity and resistance to low temperatures.

### 3.2. Female Physiological Age

All measured morphometric characteristics were in the range of the specific species’ description (Table A1, Table A2, Table A3 and Table A4). *I. ricinus* females had a lower body mass on average than the other three species (Table A1). The largest body mass was recorded in *Dermacentor* females. The highest measured body mass and the longest total body length were measured on a *D. marginatus* female, 543.40 mg and 14.198 mm, respectively (Table A2). The longest body width (alloscutal width) was observed on a *D. reticulatus* female (11.482 mm) (Table A3).

The descriptive statistics of the calculated indices and ratios are presented in Table 2. The lowest means of SI, ASR, and PAI were calculated for *I. ricinus* females. The highest SI was observed for *R. sanguineus*, but ASR and PAI for *D. marginatus*. *Dermacentor* females demonstrated similar mean SI values. However, almost equal values of PAI means were found for both *D. reticulatus* and *R. sanguineus*. Both the central tendency measure (mean) and measures of variability (V, SD, CV, and SE) had the lowest values when PAI was analyzed, which indicated higher accuracy.

**Figure A1:**
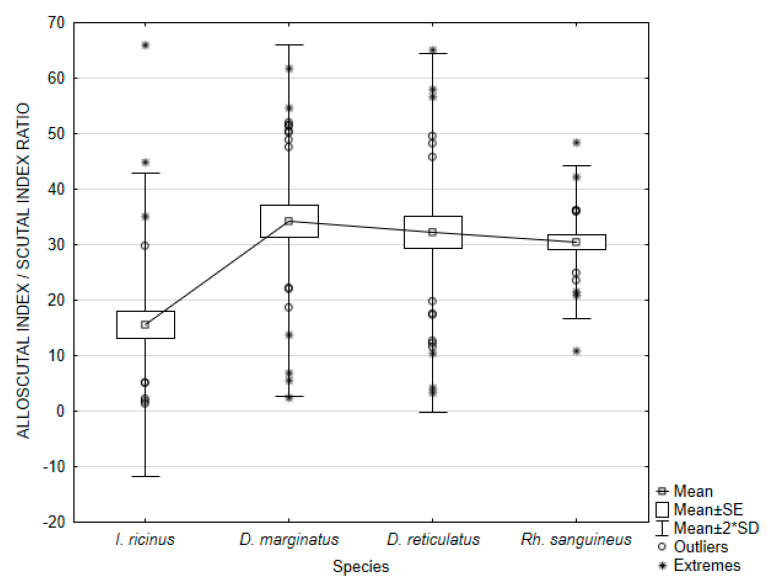
The results of Fisher’s LSD tests for calculated SI.

**Figure A2:**
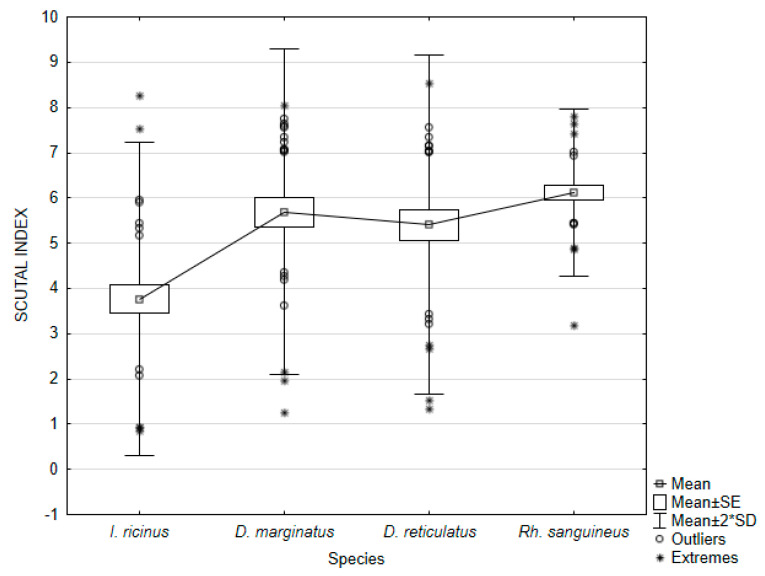
The results of Fisher’s LSD tests for calculated ASR.

**Figure A3:**
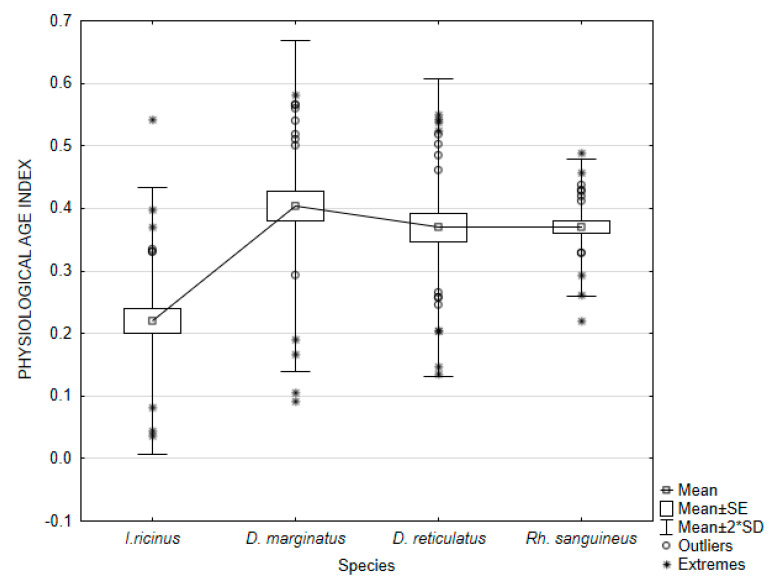
The results of Fisher’s LSD tests for calculated PAI.

One-way ANOVA emphasized high statistically significant differences when analyzing means of SI, ASR, and PAI as dependent variables and particular species as a determinant variable (*p*_SI_ = 0.000001; *p*_ASR_ = 0.000001; *p*_PAI_ = 0.000000 for *p* < 0.01). In all performed Fisher’s LSD tests, *I. ricinus* was distinguished from other species with high statistical significances (Figure A1, Figure A2 and Figure A3).

**Figure 1:**
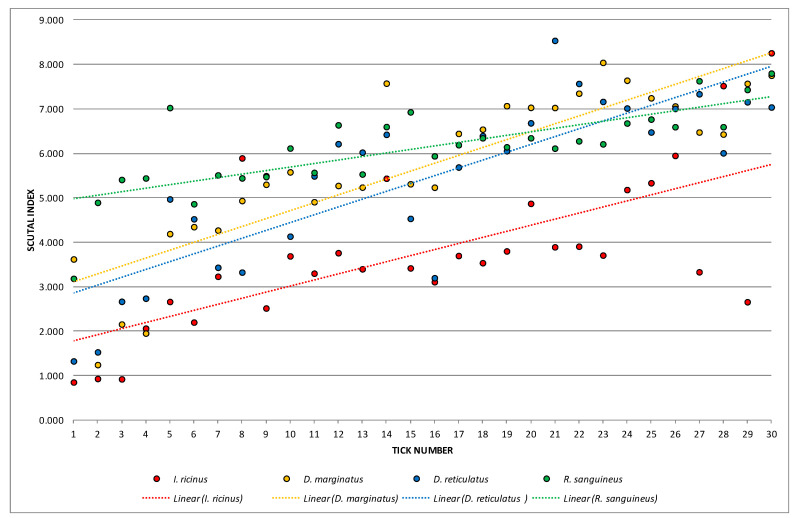
Calculated values of scutal index (SI).

**Figure 2:**
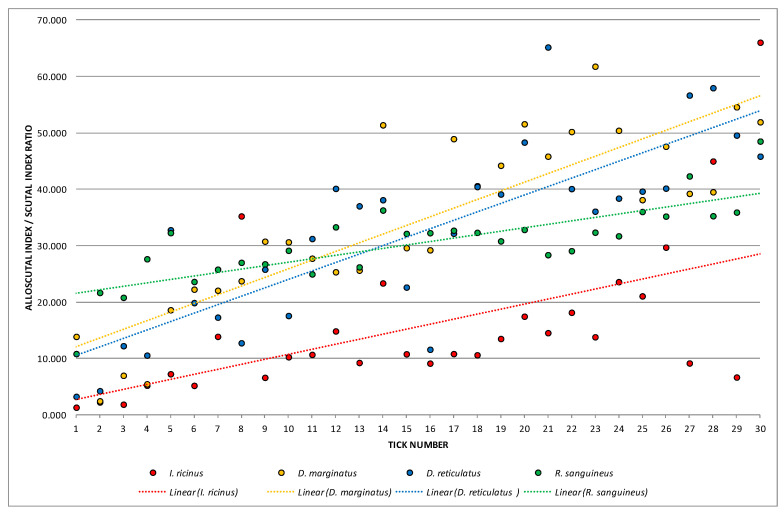
Calculated values of alloscutal index/scutal index ratio (ASR).

**Figure 3:**
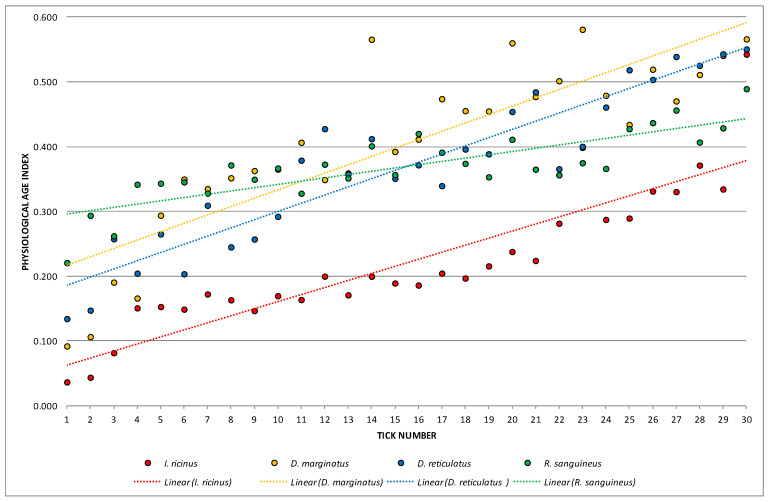
Calculated values of physiological age index (PAI).

Calculated values of SI, ASR, and PAI with linear trendlines are presented in Figure 1, Figure 2 and Figure 3. SI values were classified in nine groups (from 0.000 to 9.000) with the highest observed frequency (22.5%) in range 7 (from 7.000 to 7.9999) (Figure 1). Higher dispersion was noticed for ASR values (Figure 2). Those were arranged in seven groups (from 0.000 to 69.9999) with the highest frequency (25.0%) observed in the fourth group (20.000–39.9999). The lowest dispersion was recorded for PAI values which were separated into six groups (from 0.000–0.5999) (Figure 3). Hereby, one-third of females (33.33%) were assigned to range 4 (0.300–0.3999). Values of PAI also clearly distinguished *I. ricinus* females.

## 4. Discussion

This study was conducted in the cities geographically situated in the northern part of the Republic of Serbia, in a region characterized by a moderate continental climate. Therefore, the tick species diversity found on dogs resembles the data from other authors, especially from neighboring European countries: Hungary [6,14,32], Romania [33,34], Bulgaria [35], and Croatia [36]. Attached *Dermacentor* nymphs were not detected during this study, which is in accordance with other studies conducted on dogs [5,11,14,37]. *Dermacentor* spp. nymphs and larvae are rather nidicolous, almost exclusively endophilic parasites, which prefer burrow-dwelling and ground-living small mammals as hosts [18,38]. The results of this study also confirmed the observations of other researchers regarding the abundance increase and geographic range expansion of *D. reticulatus* in Europe [3,18,39,40,41,42]. According to the data published almost two decades ago, from 2002 to the present, *D. reticulatus* almost tripled its abundance on dogs in Serbia, from 7.55% [43] to 21.76% as recorded in this study. Nevertheless, it should be highlighted that other tick species, such as *I. ricinus* [44] and the *R. sanguineus* group [7], demonstrate a recent spread in geographical distribution in Europe as well.

Since the tick seasonal dynamics are directly influenced by climatic factors, in particular temperature, air and substrate relative humidity, the effects of global warming and climate changes certainly affect the spread, survival, and maintenance of tick populations, especially those species adapted to live in more humid habitats. Finding an adequate host(s) in order to continue the life cycle is an equally important factor for tick survival in certain habitats [45]. The presence of wild, synanthropic, and domestic animals in urban habitats has a direct impact on the tick fauna, not only from the aspect of species diversity, but also from spatial distribution and temporal dynamics. Nevertheless, we should not neglect some other, predominantly anthropogenic factors [40], such as socioeconomic changes, tourism and worldwide travelling, agricultural practices, reforestations, habitat fragmentation, grazing, pets (especially dogs) as traveling companions, hunting tourism, animal and livestock trades and transports, etc.

Knowing the tick seasonal dynamics is extremely important, since the potential risk for humans and accompanying animals to encounter ticks could be assessed and, consequently, the possible occurrence of TBD prevented. Increased abundance and appearance of ticks in certain habitats, as well as their questing activity for adequate hosts coincide with increased human and accompanying dogs’ activity in nature (sports, recreation, hunting, vacation, dog exhibitions) [46]. Our results demonstrated similar seasonal patterns in ticks found attached to dogs with those described for ticks collected from nature, especially for *I. ricinus* and two *Dermacentor* species. Numerous studies on seasonal dynamics of ixodid ticks collected from vegetation in the temperate climate regions indicate the presence of two population density peaks, in spring and autumn [47,48,49], although there is also evidence for a unimodal activity pattern in some countries [50]. *I. ricinus* and *D. reticulatus* are considered to be cold-resistant species in contrast to *D. marginatus* which is usually described as cold-sensitive and freeze-intolerant [38,41]. As regards bimodal activity, depending on the microhabitat conditions (especially temperature, relative humidity, and vegetation cover), the spring peak is clearly pronounced, while the autumn peak is slightly lower, that is especially noticeable in *I. ricinus’* case [48,51]. However, nymphs and adults of this species could be observed on vegetation at all times of the year in the temperate climatic regions [52]. The seasonal dynamics of both *Dermacentor* species were published in numerous studies in Europe [18,41,51,53,54,55].

Our results indicated that *I. ricinus* was active throughout the whole year (with the highest abundance in May) and could be found on dogs even in winter months. Although the study from Belgium confirmed this abundance peak [10], the data from Hungary highlighted its highest number in March and the absence of *I. ricinus* during December [6]. Both *Dermacentor* species were most abundant on dogs during March; however, *D. reticulatus* had another distinguished lower peak in November. Our study also confirmed the continuous winter activity of both *Dermacentor* species which was previously published [18,53,54,55]. However, these two species were not found on dogs in northern Italy during autumn and winter months [11].

According to Dantas-Torres [45], global warming certainly will not have a negative impact on the population maintenance and distribution of *R. sanguineus*, due to its specific and very adaptable life cycle. This species does not depend much on air humidity and is more resistant to dry conditions, which could additionally contribute to its geographic expansion, especially to areas where it was not detected before [45]. According to the same author, it could be predicted that an increase in average daily temperature from April to September may result in *R. sanguineus* expansion to the northern regions of Europe. As a result of its ability to easily shift from a three- to a one-host life cycle and vice versa, *R. sanguineus* is well-adapted to live and maintain high population densities within human dwellings, as well as surrounding environments, if the microclimatic conditions are favorable and suitable hosts available. Consequently, the seasonal dynamics of *R. sanguineus* collected from nature and from dogs may differ. In temperate regions, this species is most active from the late spring to early autumn [45], which is in accordance with our results. The percentage of *R. sanguineus* collected from dogs in this study (64.33%) correlates with previously published data from central Serbian regions (68.37%) [43]. However, unlike our data, in Italy, *R. sanguineus* was found on dogs throughout the year [11], with its maximum abundance in August for adults and in early September for immature stages (21.5 and 186.5, respectively) [56].

The seasonal pattern of ticks collected from dogs observed in this study followed reports for other countries, with *R. sanguineus* group as the predominant species in the spring–summer months, *D. reticulatus* typically present in winter, and adults of *I. ricinus* being generally active throughout the year [6,7,10,13,15,16,17]. This study supports the previously stated conclusions that assumptions of tick seasonality patterns on dogs should be ignored in order to prevent the increase of dog TBD [7].

In our study, *I. ricinus* had the lowest body mass values compared to other species, but still in the range that corresponded with the study of Militzer et al. [57], when an artificial feeding process was applied. Although *D. marginatus* and *D. reticulatus* are very similar, these two species differ in several characteristics [40]. *D. reticulatus*, even though slightly smaller than *D. marginatus*, is still much larger than other species of *Ixodes* genera, with fully engorged females reaching a length of over 1 cm [40], which is in accordance with our results. The average mean of *R. sanguineus* body mass in this study (134.14 mg) coincides with the results of Valim et al. [58], who studied the change in body mass of *R. sanguineus* females during artificial feeding under laboratory conditions.

According to Bartosik and Buczek [24], it is possible to determine the parasitic phase of *I. ricinus* females collected from the host, as their size and body mass change with the feeding process duration. The dorsal scutum of *I. ricinus* females is highly chitinized and does not change during the feeding period, so its length and width cannot be used directly in determining the duration of parasitism [24]. However, the scutum dimensions could be used indirectly to calculate different types of indices, such as in this study. These indices, when cautiously applied, could represent sufficiently precise parameters with very low SEs [24], which corresponds with our results. The lowest SEs were determined for PAI (from 0.009984 for *R. sanguineus* to 0.024120 for *D. marginatus*) (Table 2). Furthermore, other statistical measures of variability obtained for PAI, such as V and SD, had also the lowest values compared to the other two indices (Table 2). These results could favor PAI among all tested indices as more precise, as it had lower dispersion and, therefore, higher accuracy. Hence, SI values had the lowest CV when calculated for *I. ricinus* and *D. marginatus*.

The obtained data match the results reported by Gray et al. [30] who studied changes in morphometric parameters of *I. ricinus* nymphs and adults fed on New Zealand White rabbits (*Oryctolagus cuniculus* Linnaeus, 1758). When they could not notice the significant changes in SI in the first 24 h of feeding, these authors introduced the coxal index, i.e., the ratio of the scutum width and the distance between the fourth coxae across the ventral abdomen of the tick. The coxal index was more accurate in the first 24 h, although it was not possible to clearly differentiate recently attached ticks from those that have been feeding for 12 h. After 24 h, the changes in values of the coxal index were less accurate, and after 48 h even inapplicable, so Gray et al. [30] recommended the combined application of SI and coxal index when estimating the period of time that ticks spend on the host in order to assess the possible risk of TBD transmission. According to the same author, SI used alone cannot be used as a precise parameter to assess the risk of TBPs transmission, because, for example, *I. ricinus* can transmit *B. burgdorferi* s.l. and *B. afzelli* in a high percentage in the first 17 h after contact with the host.

In order to obtain some practical and clinically applicable tool for assessment of possible *Borrelia burgdorferi* s.l. infection after tick bite, Meiners et al. [26] investigated the SI of *I. ricinus* nymphs fed on tick-naïve Mongolian gerbils (*Meriones unguiculatus* Milne-Edwards, 1867). Interestingly, in the first 12 h of the feeding process, SI values decreased, while a slight but statistically significant increase was observed from 12 to 24 h. Furthermore, despite the significant statistical differences between the SI of ticks removed after 24 and 36 h, an overlap in values between these two groups was noted, as rapid feeders in the 24 h group had a higher SI then the slowest feeders in the 36 h group [26]. This overlap could also explain the high values of V and SD obtained for ASR in our study. As mentioned previously, ASR was calculated using only two dimensions (length and width of alloscutum and scutum), and since the duration of tick feeding on dogs was not known in this study, the overlapping and scattered values were obtained. Therefore, ASR, although not precise in our study, could provide high-quality data when determining the physiological age of unfed ticks collected from nature as proved for *I. persulcatus* [27], *I. scapularis* [29], and *I. ricinus* [59].

Calculation of PAI included body mass as a very variable three-dimensional value which stands in direct correlation with ticks’ length, width, and height (lateral width). Therefore, it had to be transformed into a linear value using the cube root [28]. Although Chaka et al. [28] applied PAI for determination of physiological age of *R. appendiculatus* larvae, nymphs, and adults fed on New Zealand White rabbits, they obtained similar results as in this study. Furthermore, the same authors emphasized that there was a positive linear correlation between the body mass of engorged nymphs and the adult tick dimensions and that PAI is highly influenced not only by scutal length and body mass but also ambient conditions, such as temperature and air humidity [28]. We could not compare these results, as the ambient conditions were not considered in our study.

## 5. Conclusions

Geographical distribution of ticks cannot be presented as a static map, but rather a dynamic system depending on numerous abiotic, biotic, and anthropogenic factors. A large number of dog owners are aware of ticks and TBDs thanks to scientific study popularization, citizen science practices, and the media. However, still, a large number of them disregard regular dog anti-tick treatments through the whole year, as they think that these notorious ectoparasites are not active during the winter months.

Since dogs serve as hosts of several tick species, tick and TBPs surveillance is unquestionably important in order to monitor and prevent the distribution of both vectors and TBDs. Additionally, we would like to emphasize the significance of the One Health approach, as subclinically infected dogs could act as a reservoir for human TBPs. Therefore, the united efforts of scientists, veterinarians, and physicians is essential in order to prevent and manage tick-borne zoonoses.

Calculation of indices based on tick morphometric characteristics is a valuable tool in the determination of tick feeding duration and, consequently, risk assessment of possible pathogen transmission. However, dog owners usually bring their dogs to the veterinary clinics after finding tick(s) already firmly attached and engorged (i.e., when they become large and visible), which indicates that it has been feeding for some time, or, which is even worse, when the first visible TBD symptoms are noticed. Therefore, it could be concluded that these and similar indices clearly have scientific value, but it is still questionable how easy they are to apply and how useful they are in everyday veterinary practice.

During the feeding process, ticks (especially females) absorb a large amount of a blood meal, which significantly modifies their morphometric, morphological, and anatomical characteristics, whereby species identification and morphometric parameters measurement require careful examination, precision, and experience. The calculated indices cannot be used as a precise method in terms of making a diagnosis or assigning adequate therapy in veterinary practice. This kind of research needs more detailed and deep studying, especially in controlled laboratory conditions where the exact duration of the feeding process can be estimated and TBP transmission detected, in order to maximally imitate the natural tick–host–pathogen system. The use of biomarkers (such as lipids) is also recommended, as, combined with tick physiological age indices, their physiology and ecology could be revealed and possible TBP transmission prevented.

## Figures and Tables

**Table 1:** The total number of collected ticks.

Species	Stage/Gender	Jan.	Feb.	Mar.	Apr.	May	June	July	Aug.	Sept.	Oct.	Nov.	Dec.	Total
*Ixodes* *ricinus*	Females	1	2	4	18	22	3	2	-	4	5	4	3	68
	Males	-	-	-	3	9	1	-	-	1	2	-	-	16
	Nymphs	-	-	-	1	5	-	-	-	-	-	-	-	6
	Total	1	2	4	22	36	4	2	0	5	7	4	3	90
*Dermacentor* *marginatus*	Females	5	6	23	5	-	-	-	-	-	1	-	1	41
	Males	-	-	14	2	-	-	-	-	-	-	-	-	16
	Nymphs	-	-	-	-	-	-	-	-	-	-	-	-	0
	Total	5	6	37	7	0	0	0	0	0	1	0	1	57
*Dermacentor* *reticulatus*	Females	2	8	45	30	20	1	-	-	-	2	38	16	132
	Males	9	4	16	16	9	-	-	-	-	-	8	6	98
	Nymphs	-	-	-	-	-	-	-	-	-	-	-	-	0
	Total	11	12	61	46	29	1	0	0	0	2	46	22	230
*Rhipicephalus* *sanguineus*	Females	-	-	-	338	30	6	9	2	1	-	-	-	386
	Males	-	-	-	175	32	3	2	1	1	-	-	-	214
	Nymphs	-	-	-	62	14	1	2	1	-	-	-	-	80
	Total	0	0	0	575	76	10	13	4	2	0	0	0	680

**Table 2:** Descriptive statistics of calculated indices and ratios.

Species	Index/Ratio	Mean	Min	Max	Variance	Standard Deviation	Coefficient of Variation	Standard Error
*Ixodes* *ricinus*	SI	3.76748	0.85284	8.25582	3.01485	1.73633	46.08731	0.317009
	ASR	15.57469	1.32172	66.03437	187.24392	13.68371	87.85861	2.498292
	PAI	0.22066	0.03657	0.54246	0.01141	0.10679	48.39508	0.019496
*Dermacentor* *marginatus*	SI	5.69296	1.24443	8.04042	3.25125	1.80312	31.67286	0.329204
	ASR	34.34066	2.45581	61.77639	249.68034	15.80128	46.01332	2.884905
	PAI	0.40394	0.09207	0.58080	0.01745	0.13211	32.70520	0.024120
*Dermacentor* *reticulatus*	SI	5.40410	1.32653	8.53510	3.50176	1.87130	34.62740	0.341651
	ASR	32.22059	3.24604	65.18115	261.39583	16.16774	50.17828	2.951812
	PAI	0.36935	0.13426	0.55036	0.01427	0.11945	32.34060	0.021809
*Rhipicephalus* *sanguineus*	SI	6.12184	3.18371	7.79968	0.85205	0.92306	15.07822	0.168528
	ASR	30.46824	10.83415	48.50751	46.82442	6.84284	22.45892	1.249325
	PAI	0.36955	0.22061	0.48910	0.00299	0.05469	14.79807	0.009984

## Data Availability

The datasets used and analyzed during the current study are available from the corresponding author upon reasonable request.

## Appendix A

Measured morphometric parameters of chosen female ticks are shown in the following tables (Table A1, Table A2, Table A3 and Table A4) with lengths and widths expressed in µm and body mass in mg.

**Table A1:** Morphometric characteristics of *Ixodes ricinus* females (lengths and widths in µm and body mass in mg).

Morphometric Characteristic	Mean	Min.	Max.	Variance	Standard Deviation	Coefficient of Variation	Standard Error
capitulum base length	211.78	172.11	282.54	984.383	31.375	14.815	5.728
capitulum base width	456.26	324.68	568.19	4023.402	63.430	13.902	11.580
length between cornua	450.54	370.83	522.58	1403.307	37.461	8.315	6.839
scutum length	1250.47	1132.63	1394.08	5167.820	71.888	5.749	13.125
scutum width	1294.79	968.93	1467.51	14,633.151	120.968	9.3433	22.086
alloscutum length	4675.89	1046.60	9678.88	4,245,321.253	2060.418	44.065	376.179
alloscutum width	3890.44	1561.61	8467.96	2,297,215.126	1515.657	38.958	276.720
lateral width	2009.09	519.41	4437.96	772,588.741	878.970	43.749	160.477
body mass	37.42	0.10	254.90	2616.720	51.154	136.714	9.339

**Table A2:** Morphometric characteristics of *Dermacentor marginatus* females (lengths and widths in µm and body mass in mg).

Morphometric Characteristic	Mean	Min.	Max.	Variance	Standard Deviation	Coefficient of Variation	Standard Error
capitulum base length	254.05	183.23	409.91	3213.630	56.689	22.314	10.350
capitulum base width	562.92	467.37	737.23	3902.831	62.473	11.098	11.406
length between cornua	425.65	326.81	535.71	2785.842	52.781	12.400	9.636
scutum length	1523.76	1136.26	1764.04	18,145.371	134.705	8.840	24.594
scutum width	1339.64	946.61	1602.67	20,545.064	143.335	10.699	26.169
alloscutum length	8640.90	2104.05	12,756.17	7,799,865.211	2792.824	32.321	509.897
alloscutum width	6696.52	2510.08	9108.71	3,117,684.124	1765.696	26.367	322.371
lateral width	4121.02	649.44	6653.48	2,151,281.139	1466.725	35.591	267.786
body mass	199.43	2.60	543.40	20,494.022	143.157	71.783	26.137

**Table A3:** Morphometric characteristics of *Dermacentor reticulatus* females (lengths and widths in µm and body mass in mg).

Morphometric Characteristic	Mean	Min.	Max.	Variance	Standard Deviation	Coefficient of Variation	Standard Error
capitulum base length	285.77	174.54	376.92	1645.662	40.567	14.196	7.286
capitulum base width	576.780	452.79	714.83	3041.439	55.149	9.562	9.905
length between cornua	479.56	326.55	630.41	3159.693	56.211	11.721	10.096
scutum length	1553.69	1283.13	1767.55	14,693.701	121.218	7.802	21.771
scutum width	1346.76	1015.76	1694.81	23,473.073	153.209	11.376	27.517
alloscutum length	8222.30	2108.31	12,198.59	7,734,051.021	2781.016	33.823	499.485
alloscutum width	6545.19	2780.21	11,482.18	3,522,816.365	1876.917	28.676	337.104
lateral width	4063.22	1356.41	7885.06	2,953,108.074	1718.461	42.293	308.645
body mass	154.10	7.80	373.70	14,924.442	122.166	79.277	21.942

**Table A4:** Morphometric characteristics of *Rhipicephalus sanguineus* females (lengths and widths in µm and body mass in mg).

Morphometric Characteristic	Mean	Min.	Max.	Variance	Standard Deviation	Coefficient of Variation	Standard Error
capitulum base length	246.67	180.68	301.37	977.779	31.269	12.677	5.709
capitulum base width	748.97	688.12	832.63	1848.157	42.990	5.739	7.849
length between cornua	386.31	313.80	451.17	1117.167	33.424	8.652	6.102
scutum length	1367.98	1198.11	1527.27	6756.647	82.199	6.009	15.007
scutum width	1368.80	1208.08	1530.67	9121.892	95.509	6.978	17.437
alloscutum length	8353.50	4862.39	11,391.66	1,601,128.141	1265.357	15.148	231.021
alloscutum width	5956.59	4283.75	7267.46	365,927.403	604.919	10.155	110.443
lateral width	3816.96	2294.94	5021.98	447,542.625	668.986	17.527	122.140
body mass	134.14	37.00	241.30	2568.976	50.685	37.784	9.254

The results of Fisher’s LSD tests for analyzed indices are presented in Figure A1, Figure A2 and Figure A3.
